# Evaluating team-based, lecture-based, and hybrid learning methods for neurology clerkship in China: a method-comparison study

**DOI:** 10.1186/1472-6920-14-98

**Published:** 2014-05-20

**Authors:** Lian-Hong Yang, Long-Yuan Jiang, Bing Xu, Shu-Qiong Liu, Yan-Ran Liang, Jin-Hao Ye, En-Xiang Tao

**Affiliations:** 1 Department of Neurology, Sun Yat-Sen Memorial Hospital of Sun Yat-Sen University, No. 107, West Yanjiang Road, Guangzhou 510120, People’s Republic of China; 2 Emergency Department, Sun Yat-Sen Memorial Hospital of Sun Yat-Sen University, No. 107, West Yanjiang Road, Guangzhou 510120, People’s Republic of China; 3 Department of Education, Sun Yat-Sen Memorial Hospital of Sun Yat-Sen University, No. 107, West Yanjiang Road, Guangzhou 510120, People’s Republic of China

**Keywords:** Lecture-based learning, Team-based learning, Neurology, Clerkship, Hybrid learning method

## Abstract

**Background:**

Neurology is complex, abstract, and difficult for students to learn. However, a good learning method for neurology clerkship training is required to help students quickly develop strong clinical thinking as well as problem-solving skills. Both the traditional lecture-based learning (LBL) and the relatively new team-based learning (TBL) methods have inherent strengths and weaknesses when applied to neurology clerkship education. However, the strengths of each method may complement the weaknesses of the other. Combining TBL with LBL may produce better learning outcomes than TBL or LBL alone. We propose a hybrid method (TBL + LBL) and designed an experiment to compare the learning outcomes with those of pure LBL and pure TBL.

**Methods:**

One hundred twenty-seven fourth-year medical students attended a two-week neurology clerkship program organized by the Department of Neurology, Sun Yat-Sen Memorial Hospital. All of the students were from Grade 2007, Department of Clinical Medicine, Zhongshan School of Medicine, Sun Yat-Sen University. These students were assigned to one of three groups randomly: Group A (TBL + LBL, with 41 students), Group B (LBL, with 43 students), and Group C (TBL, with 43 students). The learning outcomes were evaluated by a questionnaire and two tests covering basic knowledge of neurology and clinical practice.

**Results:**

The practice test scores of Group A were similar to those of Group B, but significantly higher than those of Group C. The theoretical test scores and the total scores of Group A were significantly higher than those of Groups B and C. In addition, 100% of the students in Group A were satisfied with the combination of TBL + LBL.

**Conclusions:**

Our results support our proposal that the combination of TBL + LBL is acceptable to students and produces better learning outcomes than either method alone in neurology clerkships. In addition, the proposed hybrid method may also be suited for other medical clerkships that require students to absorb a large amount of abstract and complex course materials in a short period, such as pediatrics and internal medicine clerkships.

## Background

Students deal with a large number of diverse nervous system disorders in clinical situations and perceive neurology as being overly complex, abstract, and far more difficult than other disciplines [1-3]. At the same time, bridging medical theory and clinical practice, the clerkship is one of the crucial stages in neurology education; it develops students’ clinical reasoning and practical skills [4]. However, the traditional lecture-based learning (LBL) widely used in neurology clerkship education often leads to unsatisfactory learning outcomes because medical students passively receive knowledge from instructors with little interaction, and lack motivation to study and innovate [5,6]. It is important to develop a practical and effective learning method for neurology clerkship education [7,8].

Team-based learning (TBL), as proposed by Michaelsen et al. [9] at the University of Oklahoma, is an instructional strategy that aims to improve students’ teamwork spirit and cooperation skills. TBL requires students to read the course materials and do preliminary homework prior to their class, and encourages them to work together effectively as a team to solve problems during the class. Before the recent introduction of TBL to medical education, it was implemented in other educational curricula for many years. TBL has been successfully used in the education of basic medical sciences such as physiology and anatomy [10-14], and was widely accepted by students [15,16]. Despite its success in teaching basic sciences, we feel that TBL has its drawbacks when applied to neurology clerkship education. TBL relies on students to prepare and do their homework by themselves, but the neurology course materials are usually very difficult for them to understand, and it is even harder for students to build a clear and well-organized knowledge hierarchy. Meanwhile some research also found that TBL may benefit only students who are less able to learn and need to be helped by other team members [17,18], or only those with excellent innovative thinking [19]. These drawbacks motivated us to explore a more effective method of neurology clerkship training.

According to the literature and our experiences, LBL excels in breaking down difficult subjects by logically organizing them in a clear hierarchy and presenting them systematically. Conversely, TBL excels in motivating students to learn proactively and promotes team collaboration. The strengths of each method may complement the weaknesses of the other. Combining TBL with LBL may produce better learning outcomes than TBL or LBL alone.

In this study, we proposed a new hybrid LBL and TBL method (TBL + LBL) for neurology clerkship education. In addition, we designed an experiment to teach three groups of students with LBL, TBL, and TBL + LBL, respectively. We compared the theoretical and practice test scores of these three groups at the end of the experiment to evaluate the TBL + LBL learning outcomes. In addition, a questionnaire was completed by the students in the TBL and TBL + LBL groups to evaluate their satisfaction.

## Methods

This study on neurology clerkship education was organized by the Department of Neurology at Sun Yat-Sen Memorial Hospital in a five-year undergraduate program for students majoring in Clinical Medicine. The fourth year of the program is dedicated to clerkships in many medical specialties, including two weeks in neurology. The entire 127-student class of Grade 2007, Department of Clinical Medicine, Zhongshan School of Medicine, Sun Yat-Sen University (SYSU) participated in this neurology clerkship.

Although the students were given the opportunity not to participate in this study, all students chose to participate. The students were randomly assigned to one of three groups by drawing lots: Group A (TBL + LBL, with 41 students including 22 men and 19 women), Group B (LBL, with 43 students including 23 men and 20 women), and Group C (TBL, with 43 students including 21 men and 22 women). Before the clerkship started, all students had completed the related courses. No significant difference was observed between the three groups in terms of sex, age, and theoretical test score before the program. The program was organized into 2-week sessions for each group, which were held one after another in the order of Groups B, C, and A. Each group spent the same amount of time studying with the instructor.

## LBL method

The curriculum consisted of many case studies that covered several important neurology courses, such as Physical Examination of Nervous System, Nervous System Qualitative and Positioning Diagnosis Principles, Principles for Differential Diagnosis, Cranial Neuropathy and Peripheral Neuropathy, Cerebrovascular Diseases, Spinal Cord Diseases, and Peripheral Nervous System Diseases. For each type of case, all Group B students were led into a big room dedicated for teaching in the Department of Neurology of the hospital. The patients were temporarily moved to this room before the demonstration. The instructor first demonstrated the process of medical history inquiry and physical examination, and then provided the examination results to the students as a reference. After the demonstration, the instructor gave lectures to explain the clinical characteristics of the cases, the special examination methods for the cases, the key points of disease identification, diagnosis, and treatment, as well as the fundamental concepts for many common neurological diseases. These common neurological diseases include cerebrovascular, spinal cord, and peripheral nervous system diseases. Subsequently, the students were required to identify the characteristics of each case and propose a diagnosis and treatment plan before the instructor summarized the case study and drew conclusions for the students.

## TBL method

Group C participated in the second clerkship session. Compared with Group B, the instructor, the curriculum, the learning objectives, and the time spent with the instructor were all the same. The only difference was in the learning methods. As for Group B, the instructor conducted a similar demonstration at the beginning of each case study. However, the groups diagnosed different patients because the majority of patients usually stayed for less than two weeks at the hospital and therefore it was difficult to use the same patients for case diagnosis in separate 2-week sessions. Nevertheless, we carefully chose the patients for the same cases so that their disease types, symptoms, and diagnosis results were similar, to minimize the difference in learning experiences. After the demonstration, instead of receiving lectures as Group B did, the students in Group C studied the same topics in a team-based way as described below.

Group C were divided into seven teams with each comprising five to seven students. Following a process described previously [20], TBL was conducted in three phases. Phase One was a preparatory phase. The students first familiarized themselves with the course objectives, requirements, and the case information given by the instructor prior to the class. On this foundation, the students studied relevant materials using a range of resources including textbooks, libraries, and the Internet. Team members collaborated with each other, but they also focused on different aspects ranging from clinical characteristics, examinations, diagnosis to treatment, respectively. Phase Two was a readiness assurance process, including an individual readiness assessment test (iRAT) and a group readiness assessment test (gRAT). Both tests were closed-book. The iRAT contained 10 multiple-choice questions and required each student to give the answers within 20 minutes. The questions were pertinent to the case and focused on fundamental concepts, clinical characteristics, and examinations. The gRAT was performed after the patient had been admitted to the hospital and the detailed examination results had become available. It included 10 multiple-choice questions for the teams to answer within 30 minutes. The questions focused on disease identification, diagnosis, and treatment choice. At first only intrateam discussions were allowed, through which each team was required to obtain its own answers. Next, interteam discussions and debates were performed to identify a set of answers that all students accepted. Afterwards, the instructor evaluated the discussions and the answers, and provided feedback accordingly to help the students achieve a clearer and more systematic understanding of the required knowledge and skills. Phase Three was the application of course concepts. Considering that students had acquired the necessary knowledge and skills through phases One and Two, the instructor presented five to eight complex questions. The questions required students to summarize, analyze, and reason carefully based on the knowledge they had learnt and the cases they had observed. Similarly to the process in Phase Two, intrateam studies and discussions took place first. Afterwards, interteam discussions and debates were performed between the teams holding different opinions. The instructor then commented on each team’s discussion and summarized the main topics of the course. As the last step, the students were encouraged to evaluate each other based on their performance, in terms of motivation, analytical skills, and expression skills.

### TBL + LBL method

Both TBL and LBL were employed by Group A. During the first week, Group A received LBL, which covered complex and systematic courses such as Physical Examination of Nervous System, Nervous System Qualitative and Positioning Diagnosis Principles, Principles for Differential Diagnosis, as well as Cranial Neuropathy and Peripheral Neuropathy. The procedure was similar to the one used for Group B. During the second week, TBL was employed for three common neurological courses: Cerebrovascular Diseases, Spinal Cord Diseases, and Peripheral Nervous System Diseases. The procedure was similar to the one used for Group C. Among the three groups, the curriculum, the instructor, the learning objectives, and the time spent with the instructor were all identical.

### Performance and satisfaction evaluation

At the end of the clerkship, the performance of each student was evaluated with a theoretical test and a practice test. Well established in the medical program, the test content for the three groups was identical. The theoretical test mainly evaluated the students’ understanding of the fundamental concepts, causes, characteristics, diagnosis, and treatment for the diseases covered in the clerkship. The test questions were carefully selected from the standard question pool at SYSU. The scores of this test were not comparable with those of the preclerkship test because the two tests have different levels of difficulty. The practice test mainly evaluated the students’ proficiency of conducting disease history inquiry, physical examination, and medical record writing. Each student was rated with a hundred-mark weighted total score, 40% of which was based on the practice test score and 60% was based on the theoretical test score. The weighting followed the teaching guidelines of SYSU and had been used consistently for many years. In addition to the tests, Groups A and C were required to complete a questionnaire to evaluate their satisfaction with TBL. The satisfaction was evaluated for various aspects in a four-point scale: excellent, good, fair, and poor. The answers were collected at the scene.

### Data analysis

All analyses were conducted with SPSS software (version 13.0; SPSS, Chicago, IL, USA). Enumeration data collected were analyzed using *χ*^2^ test. Measurement data were expressed as mean ± standard deviation, and *t*-test was conducted between the two groups. The level of significance was set at 0.05.

### Ethics approval

The local Institutional Review Board at the Sun Yat-Sen Memorial Hospital of SYSU waived ethics approval (application number: 20120928) for this research because the study protocol was not deemed to represent biomedical or epidemiological research and no personal data were used. The procedures complied with data-protection rules, and all data were anonymized prior to analysis.

## Results

### Base level of the students in the three groups

The three groups consisted of only fourth-year students. As shown in Table 1, no significant difference was observed between the three groups in terms of student numbers, sex, age, and preclerkship theoretical test score.

**Table 1:** Baseline of the students in the three groups

**Group**	**Number of students (*n*)**	**Sex ratio (male/female)**	**Age**^**†**^	**Theoretical test scores before the clerkship**^**†**^
A (TBL + LBL)	41	22/19	21.4 ± 2.81*	83.9 ± 8.33*
B (LBL)	43	23/20	21.6 ± 2.25	84.2 ± 7.59
C (TBL)	43	21/22	22.0 ± 1.76	84.7 ± 6.97

*Compared with Groups B and C, *P* > 0.05, which means the differences between Group A and the other groups are insignificant.

^†^Expressed as mean ± standard deviation.

### Comparison at the end of the clerkship

**Figure 1:**
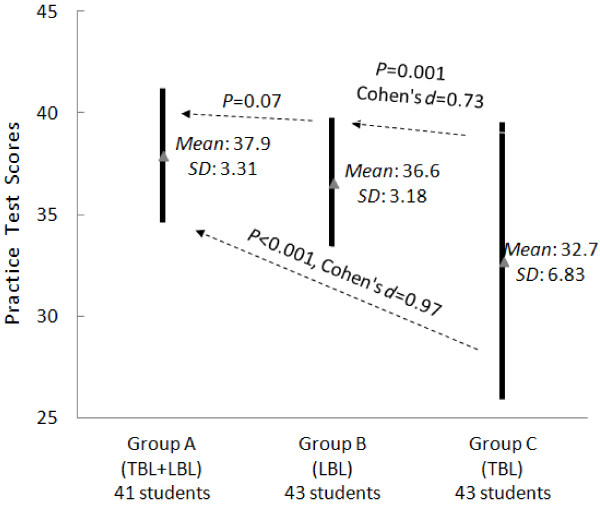
**Comparison of the practice test scores at the end of the clerkship.** The test scores of each group are represented by a thick solid line. The comparison between the test scores of every two groups is explained with a comment on a dotted line. In the comment for each solid line, *Mean* stands for the mean score of the corresponding group, while *SD* stands for the standard deviation. Each solid line centers on *Mean* and stretches from (*Mean – SD*) to (*Mean* + *SD*). The longer the solid line is, the bigger the *SD* the corresponding test scores have. For each dotted line, its comment states the *t*-test result. If the result indicates significant difference (*P* < 0.05), the calculated effect size is also presented. The effect size can be either positive or negative. A positive effect size indicates positive difference in the direction of the arrow.

**Figure 2:**
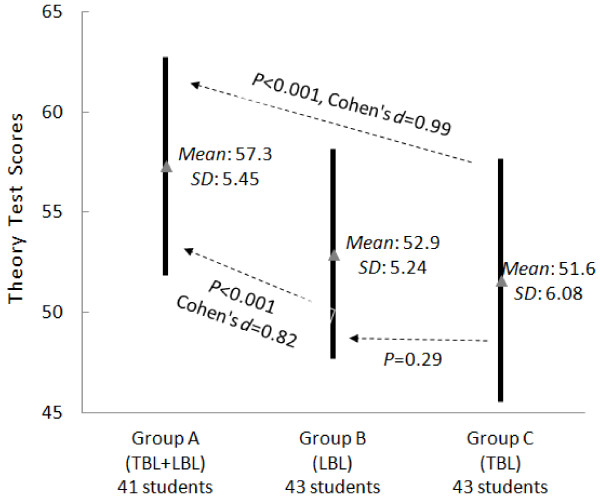
**Comparison of the theoretical test scores at the end of the clerkship.** Please refer to the legends of Figure 1 for the meanings of solid lines, dotted lines, and their comments.

**Figure 3:**
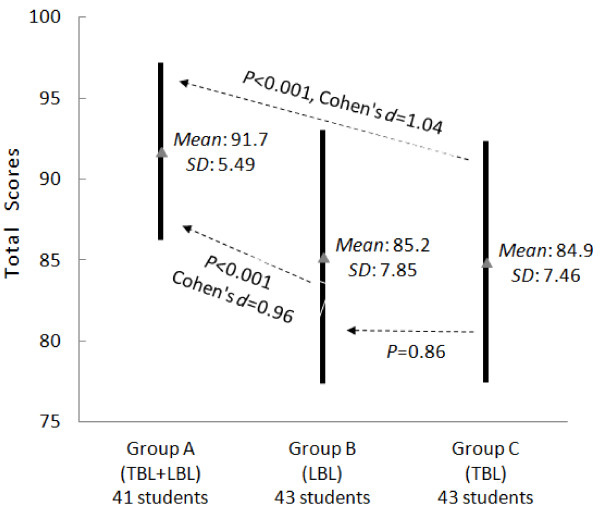
**Comparison of the total scores at the end of the clerkship.** Please refer to the legends of Figure 1 for the meanings of solid lines, dotted lines, and their comments.

Analysis of the test scores at the end of the clerkship is illustrated in Figures 1, 2, and 3. Group A achieved the highest scores. Groups A and B achieved similar practice test scores, but Group A achieved higher theoretical test and total scores (*P* < 0.05). Compared with Group C, Group A had higher scores in all aspects (*P* < 0.05). No significant difference between Groups B and C was observed in terms of theoretical test scores and total scores. However, Group C had much lower practice test scores than did Group B.

### Questionnaire

The response rate to the questionnaire was 100%. The content of the questionnaire and the results are shown in Table 2. At least 85% of students had positive feedback for TBL. They regarded TBL as an innovative learning method and agreed that it created an active classroom atmosphere in the neurology clerkship courses, enhanced learning motivation, strengthened teamwork spirit, and improved their ability to solve real clinical problems. In summary, TBL was highly accepted by the majority of students. Conversely, some students had negative feedback on TBL, which was mainly focused on the fact that the teaching topics were less structured and less systematically organized, and thus it was more difficult for the students to gain a comprehensive understanding of the subjects. The students agreed that LBL could help them learn the relatively complex and nonintuitive parts more easily than TBL. By contrast, TBL + LBL achieved 100% satisfaction, as shown in Table 2.

**Table 2:** Questionnaire results from students in group A (TBL + LBL) and Group C (TBL) (number of responses (%))

**Aspects**	**Excellent**	**Good**	**Fair**	**Poor**
Increasing motivation to prepare before class	70 (83.3)	7 (8.3)	5 (6.0)	2 (2.4)
Increasing motivation and ability of thinking	71 (84.5)	7 (8.3)	4 (4.8)	2 (2.4)
Activating class atmosphere	79 (94.0)	3 (3.6)	2 (2.4)	0 (0)
Promoting teamwork spirit and ability	76 (90.5)	5 (6.0)	3 (3.5)	0 (0)
Developing clinical problem-solving skills	66 (78.6)	6 (7.1)	8 (9.5)	4 (4.8)
Improving the understanding of important and difficult topics	60 (71.4)	7 (8.3)	8 (9.5)	9 (10.7)
Satisfaction with pure TBL	67 (79.8)	6 (7.1)	7 (8.3)	4 (4.8)
Satisfaction with TBL + LBL^*^	36 (87.8)	5 (12.2)	0 (0)	0 (0)

*Only Group A was required to answer.

## Discussion

In summary, our experiment led to three findings. First, the practice test scores of Group A (TBL + LBL) were similar to those of Group B (LBL), but significantly higher than those of Group C (TBL) were. In other words, the groups using LBL performed better in the practice test. Second, the theoretical test scores and the total scores of Group A were significantly higher than those of Groups B and C. Last, the questionnaire showed that the hybrid method was widely accepted by the students.

Why did the two learning methods involving LBL lead to better practice test scores? The reason is twofold. First, LBL is suitable for neurology, which is known to be less accessible and user-friendly than other specialties [2]. In LBL, lectures break down the difficult subjects of neurology into small topics, logically organize them in a relatively clear hierarchy, and present them systematically. This method is easier for students to memorize and understand the information presented. LBL also helps students connect and transform simple and abstract knowledge into a concrete, logical, and comprehensive perspective. Second, LBL lays emphasis on developing fundamental clinical skills such as effective and accurate disease history inquiry, comprehensive nervous system examination, proper and formal medical record writing, as well as good communication between doctors and patients. These are all essential skills in clinical practice. Therefore, although TBL offers many advantages, traditional LBL is still indispensable for neurology clerkship education. Conversely, LBL also has disadvantages. For instance, in our study we noticed that the students in the LBL groups tended to learn passively without proactive preparation before classes or review after classes. We also felt that they were less able to concentrate in the classroom and less motivated to take advantage of various learning resources. Other researchers have found that LBL is insufficient for the absorption of the culture of clinical thinking [21] and teamwork spirit [9,11].

Combining LBL with TBL is an effective way to overcome these disadvantages of LBL. TBL stresses the encouragement of students to learn proactively as well as to improve their analytical and problem-solving skills in clinical situations. Moreover, TBL is known to be beneficial for promoting teamwork and collaboration between students [9,14,20]. At the same time, TBL implicitly enhances the students’ skills in bibliographic retrieval, logical reasoning, and oral presentation. These key abilities are extensively required in clinical practice, but often less practiced in LBL. The questionnaire results revealed that the majority of students accepted TBL and agreed that TBL could result in an active classroom atmosphere, enhance their motivation to learn, strengthen teamwork spirit, as well as improve their clinical problem-solving skills.

Furthermore, instructors can improve their teaching expertise during the TBL because successful TBL requires that they prepare questions with a certain level of difficulty related to the course content and the specific clinical diagnosis and treatment processes. This motivates the instructors to acquire not only the necessary solid knowledge and clinical skills, but also skills in organizing and managing people. In addition, instructors may also be inspired by team discussions. In summary, TBL can be of value to both teachers and learners [22,23].

However, we observed several disadvantages of TBL that were also reported by others [24]. First, TBL is less effective compared with LBL in students with weak self-directed study abilities. This ineffectiveness produces larger variation in student learning outcomes. Second, for TBL to achieve better learning outcomes than LBL, students require a solid understanding of related neurology theories. Otherwise, it is very difficult for the students to understand abstract neurology concepts and engage in effective discussion. To support this finding, our results show that because the TBL groups did not receive as much systematic training in the classroom as the LBL groups did, they did not achieve significantly better scores than the LBL group (Figures 1, 2 and 3). Last, compared with LBL, TBL students often have less capability to learn knowledge in a systematic way. The knowledge learnt with TBL tends to be not as deep or as comprehensive as with LBL. For instance, based on the feedback from the students in the TBL group, they tended to miss some important topics.

Based on our experiences with TBL + LBL, we feel that the instructors play a very important role and they need to be trained specifically. This training has three goals: 1) to design questions and cases closely related to the clinical practice, which should have clear emphasis and appropriate difficulty; 2) to facilitate learning by encouraging students as well as providing ample practice opportunities for them to develop clinical skills; 3) to summarize and conclude after each topic for students to gain complete and in-depth understanding of the knowledge supplied.

There were two limitations for this research. First, because no questionnaire was completed by the LBL groups, the authors do not know what the LBL groups thought of their learning experiences, so no comparisons can be made on student perspective. Though the results of the questionnaire indicated that Groups A and C had experienced increased motivation and teamwork spirit, the basis of comparison was their past LBL experiences in other subjects. Second, there may be a possibility of bias in student self-reports on the evaluation questionnaire. To minimize this bias, the questionnaire was sent to the students only after the theoretical and the practice tests were completed. We will improve these in further research.

## Conclusions

Our results support our proposal that combining TBL and LBL is acceptable to students and produces better outcomes for learning than either method alone in neurology clerkship education. In addition, the proposed hybrid method may also be suited for other medical clerkships that require students to learn large amount of abstract and complex course materials in a short period, such as clerkships in pediatrics or internal medicine. The processes for all types of clerkships are similar. LBL can be applied to the relatively complex and systematic courses while TBL can be applied to others. Moreover, the method can also be applied in other universities and countries. However, for optimal learning outcomes, the time and the courses assigned to each method should be carefully determined based on the course contents and students’ characteristics. How to optimally design the courses based on these factors is a valuable research topic for further study.

## Abbreviations

TBL: Team-based learning; LBL: Lecture-based learning; TBL + LBL: Hybrid team- and lecture-based learning; SYSU: Sun Yat-Sen University; iRAT: Individual readiness assessment test; gRAT: Group readiness assessment test.

## Competing interests

The authors declare that they have no competing interests.

## Authors’ contributions

LHY and LYJ contributed equally to this article. They analyzed the characteristics of neurology clerkship education, proposed the TBL + LBL method, designed the experiment and evaluation method accordingly, and played a leading role in writing the article. BX arranged the course content for the clerkship program and helped design the teaching procedure for the three groups. SQL designed and carried out the questionnaire, summarized the results, and provided analysis results. YRL collected and analyzed the data. She also played an important role in writing and editing the article. JHY helped design and carry out the theoretical test and contributed to the design and implementation of the iRAT and gRAT tests. EXT helped design and carry out the practice test and contributed to the design and implementation of the iRAT and gRAT tests. All authors read and approved the final manuscript.

## Pre-publication history

The pre-publication history for this paper can be accessed here:

http://www.biomedcentral.com/1472-6920/14/98/prepub

## References

[B1] Ridsdale L Massey R Clark L Preventing neurophobia in medical students, and so future doctors Pract Neurol 2007 7 116 123 17430877

[B2] Schon F Hart P Fernandez C Is clinical neurology really so difficult? J Neurol Neurosurg Psychiatry 2002 72 557 559 10.1136/jnnp.72.5.557 11971033 PMC1737866

[B3] Flanagan E Walsh C Tubridy N’Neurophobia’– attitudes of medical students and doctors in Ireland to neurological teaching Eur J Neurol 2007 14 1109 1112 10.1111/j.1468-1331.2007.01911.x 17880566

[B4] Menken M Hopkins A Walton H Statement on medical education in neurology Med Educ 1994 28 271 274 10.1111/j.1365-2923.1994.tb02711.x 7861996

[B5] Norman G Research in clinical reasoning: past history and current trends Med Educ 2005 39 418 427 10.1111/j.1365-2929.2005.02127.x 15813765

[B6] Enarson C Cariaga-Lo L Influence of curriculum type on student performance in the United States medical licensing examination step 1 and step 2 exams: problem-based learning vs. lecture-based curriculum Med Educ 2001 35 1050 1055 10.1046/j.1365-2923.2001.01058.x 11703641

[B7] Menken M Demystifying neurology: phenomenology can help BMJ 2002 324 1469 1470 10.1136/bmj.324.7352.1469 12077019 PMC1123429

[B8] Lim EC Seet RC Demystifying neurology: preventing ‘neurophobia’ among medical students Nat Clin Pract Neurol 2008 4 462 463 18594504 10.1038/ncpneuro0849

[B9] Michaelsen LK Knight AB Fink LD Team-based learning: a transformative use of small groups 2002 Westport: Greenwood Publishing Group 157 171

[B10] Haidet P Morgan RO O’Malley K Moran BJ Richards BF A controlled trial of active versus passive learning strategies in a large group setting Adv Health Sci Educ 2004 9 15 27 10.1023/B:AHSE.0000012213.62043.45 14739758

[B11] Kelly PA Haidet P Schneider V Searle N Seidel CL Richards BF A comparison of in-class learner engagement across lecture, problem-based learning, and team learning using the STROBE classroom observation tool Teach Learn Med 2005 17 112 8 10.1207/s15328015tlm1702_4 15833720

[B12] Searle NS Haidet P Kelly PA Schneider VF Seidel C Richards BF Team learning in medical education: Initial experiences at ten institutions Acad Med 2003 78 S55 8 10.1097/00001888-200310001-00018 14557096

[B13] Nieder GL Parmelee DX Stolfi A Hudes PD Team-based learning in a medical gross anatomy and embryology course Clin Anat 2005 18 56 63 10.1002/ca.20040 15597377

[B14] Thompson BM Schneider VF Haidet P Levine RE McMahon KK Perkowski LC Richards BF Team-based learning at ten medical schools: two years later Med Educ 2007 41 250 257 10.1111/j.1365-2929.2006.02684.x 17316209

[B15] Tai BC Koh WP Does team learning motivate students’ engagement in an evidence-based medicine course? Ann Acad Med Singapore 2008 37 1019 1023 19159035

[B16] Thomas PA Bowen CW A controlled trial of team-based learning in an ambulatory medicine clerkship for medical students Teach Learn Med 2011 23 31 36 10.1080/10401334.2011.536888 21240780

[B17] Koles P Nelson S Stolfi A Parmelee D DeStephen D Active learning in a year 2 pathology curriculum Med Educ 2005 39 1045 55 10.1111/j.1365-2929.2005.02248.x 16178832

[B18] Vasan NS DeFouw D Team learning in a medical gross anatomy course Med Educ 2005 39 524 15842708 10.1111/j.1365-2929.2005.02146.x

[B19] Mayer RE Applying the science of learning to medical education Med Educ 2010 44 543 549 10.1111/j.1365-2923.2010.03624.x 20604850

[B20] Michaelsen L Knight AB Fink LD Team-based learning: A transformative use of small groups in college teaching 2004 Sterling (VA): Stylus Publishing

[B21] Michaelsen LK Parmelee DX McMahon KK Levine RE Billings DM Team-Based Learning for Health Professions Education: A guide to Using Small Groups for Improving Learning 2008 Sterling (VA): Stylus Publishing 14

[B22] Parmelee DX Michaelsen LK Twelve tips for doing effective team-based learning (TBL) Med Teach 2010 32 118 122 10.3109/01421590903548562 20163226

[B23] Michaelsen L Richards B Drawing conclusions from the team-learning literature in health-sciences education: a commentary Teach Learn Med 2005 17 85 8 10.1207/s15328015tlm1701_15 15691820

[B24] Gao X Ma W TBL (Team Based Learning) teaching model in education of surgery Researches in Medical Education 2010 9 9 1230 1231

